# A Nanomedicine Fabricated from Gold Nanoparticles‐Decorated Metal–Organic Framework for Cascade Chemo/Chemodynamic Cancer Therapy

**DOI:** 10.1002/advs.202001060

**Published:** 2020-06-14

**Authors:** Yuan Ding, Hao Xu, Chang Xu, Zongrui Tong, Sitong Zhang, Yang Bai, Yining Chen, Qianhui Xu, Liuzhi Zhou, Hao Ding, Zhongquan Sun, Sheng Yan, Zhengwei Mao, Weilin Wang

**Affiliations:** ^1^ Department of Hepatobiliary and Pancreatic Surgery the Second Affiliated Hospital School of Medicine Zhejiang University Hangzhou Zhejiang 310009 China; ^2^ Key Laboratory of Precision Diagnosis and Treatment for Hepatobiliary and Pancreatic Tumor of Zhejiang Province Hangzhou Zhejiang 310009 China; ^3^ Research Center of Diagnosis and Treatment Technology for Hepatocellular Carcinoma of Zhejiang Province Hangzhou Zhejiang 310009 China; ^4^ Clinical Medicine Innovation Center of Precision Diagnosis and Treatment for Hepatobiliary and Pancreatic Disease of Zhejiang University Hangzhou Zhejiang 310009 China; ^5^ Clinical Research Center of Hepatobiliary and Pancreatic Diseases of Zhejiang Province Hangzhou Zhejiang 310009 China; ^6^ MOE Key Laboratory of Macromolecular Synthesis and Functionalization Department of Polymer Science and Engineering Zhejiang University Hangzhou Zhejiang 310027 China; ^7^ Key Laboratory of Precision Diagnosis and Treatment for Hepatobiliary and Pancreatic Tumor of Zhejiang Province Hangzhou Zhejiang 310009 China

**Keywords:** cascade reactions, chemodynamic therapy, nanomedicine, nanozyme, synergistic efficacy

## Abstract

The incorporation of new modalities into chemotherapy greatly enhances the anticancer efficacy combining the merits of each treatment, showing promising potentials in clinical translations. Herein, a hybrid nanomedicine (**Au/FeMOF@CPT NPs**) is fabricated using metal–organic framework (MOF) nanoparticles and gold nanoparticles (Au NPs) as building blocks for cancer chemo/chemodynamic therapy. MOF NPs are used as vehicles to encapsulate camptothecin (CPT), and the hybridization by Au NPs greatly improves the stability of the nanomedicine in a physiological environment. Triggered by the high concentration of phosphate inside the cancer cells, **Au/FeMOF@CPT NPs** effectively collapse after internalization, resulting in the complete drug release and activation of the cascade catalytic reactions. The intracellular glucose can be oxidized by Au NPs to produce hydrogen dioxide, which is further utilized as chemical fuel for the Fenton reaction, thus realizing the synergistic anticancer efficacy. Benefitting from the enhanced permeability and retention effect and sophisticated fabrications, the blood circulation time and tumor accumulation of **Au/FeMOF@CPT NPs** are significantly increased. In vivo results demonstrate that the combination of chemotherapy and chemodynamic therapy effectively suppresses the tumor growth, meantime the systemic toxicity of this nanomedicine is greatly avoided.

## Graphical abstract

A hybrid nanomedicine (Au/FeMOF@CPT NPs) consisting of metal–organic framework nanoparticles (MOF NPs) and Au NPs is developed for cancer chemo/chemodynamic therapy. MOF NPs are used as vehicles to encapsulate camptothecin (CPT). H_2_O_2_ from the oxidation of intracellular glucose by Au NPs is further utilized as fuel for the Fenton reaction, thus realizing the synergistic anticancer efficacy.

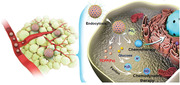

Chemotherapy, a therapeutic modality utilizing highly toxic drugs to kill cancer cells, has played a pivotal role in cancer treatment over the past decades.^[^ ^1^ ^]^ However, poor drug delivery, unsatisfactory antitumor performance, severe side effects, and drug resistance greatly impair their clinical outcome.^[^ ^2^ ^]^ Benefit from nanotechnology, effective delivery of chemotherapeutic drugs is achieved by the enhanced permeability and retention (EPR) effect, which prolongs their circulation time and enhances the tumor‐specific accumulation.^[^ ^3^ ^]^ Among various nanomedicine systems, metal–organic framework (MOF) nanoparticles have unique advantages beyond drug loading and controlled delivery based on tailor‐designed porous structure.^[^ ^1^, ^4^ ^]^ The presence of metal ions and organic ligands endows the resultant MOF platforms with excellent theranostic capability. For example, chemodynamic therapy (CDT) that catalytically converts intracellular hydrogen dioxide (H_2_O_2_) into highly toxic reactive oxygen species (ROS) through therapeutic reactions, especially Fenton‐like reactions, can be integrated into the MOF nanoparticles to promote the anticancer performance.^[^ ^5^ ^]^ Various studies have demonstrated that the nanomedicines combining chemotherapy and CDT exhibit excellent synergy effect in suppressing tumor growth and inhibiting tumor metastasis.^[^ ^5^, ^6^ ^]^

Unfortunately, insufficient endogenous H_2_O_2_ in the tumor tissue significantly restricts the further applications of CDT.^[^ ^7^ ^]^ The existed strategies such as directly encapsulating exogenous H_2_O_2_ are facing several serious issues, such as the inevitable damage to normal tissues caused by the leakage of H_2_O_2_ from carriers.^[^ ^8^ ^]^ Glucose oxidase (GOx) with the ability to produce H_2_O_2_ by catalyzing glucose meets the requirement of H_2_O_2_ generation in situ. However, the uncontrollable reaction between GOx and glucose during delivery processes and relative low operational stability of GOx hamper the practical in vivo application of this strategy.^[^ ^5^, ^9^ ^]^

Therefore, developing nanoformulations that can specifically and efficiently catalyze glucose to H_2_O_2_ transforming will achieve the purpose of precisely killing cancer cells, together with MOF‐based nanomedicine. Fortunately, gold nanoparticles (Au NPs) with small size exhibit highly efficient and stable GOx‐mimic catalytic activity, which can oxidize intracellular glucose to produce H_2_O_2_.^[^ ^10^ ^]^ More importantly, the catalytic activity of Au NPs can be rationally controlled by their surface hydrophobicity and subsequent interaction with water soluble glucose, enabling to specifically activate their enzyme‐mimic catalytic capability in cancer cells, thus solving tricky issues faced by the natural GOx for cancer treatments.

Herein, we prepare a sophisticated nanomedicine with cascade catalytic capability for cancer chemo/chemodynamic therapy on the basis of a hybridization strategy (**Scheme** **1**). Using iron(III) *meso*‐tetra(4‐carboxyphenyl)porphine chloride **TCPP(Fe)** and zirconyl cluster as building blocks, MOF nanoparticles (**FeMOF** NPs) are fabricated that are further utilized as platforms to construct the hybrid composite by in situ growth of small Au NPs on the surface. **FeMOF** NPs with porous structure can be used to encapsulate a hydrophobic chemotherapeutic drug (camptothecin, CPT) and the anchored Au NPs can be further modified by 1‐dodecanethiol (**C_12_SH**) and methoxy polyethylene glycol thiol (**PEG‐SH**) to improve the stability of the nanomedicine and to block the catalysis during circulation. Interestingly, the nanohybrids completely collapse after cellular internalization triggered by the intracellular phosphate due to its stronger coordination with zirconium, thus boosting the drug release and activating the cascade catalytic reactions. Au NPs act as artificial enzyme to oxidize the intracellular glucose to elevate the endogenous H_2_O_2_ level, which is the chemical fuel for Fenton reaction catalyzed by **TCPP(Fe)** to produce highly active OH∙. It should be emphasized that the consumption of glucose also contributes to the anticancer efficacy through a starvation strategy. Attributing to smart nanotechnology and the EPR effect, the circulation time of **PEG‐Au/FeMOF@CPT NPs** is remarkably prolonged and its tumor accumulation is greatly enhanced, facilitating to improve therapeutic efficacy and avoid systemic toxicity. In vitro and in vivo studies verify the satisfactory therapeutic performance of this hybrid nanomedicine, significantly inhibiting the tumor growth combining chemotherapy and chemodynamic therapy.

**Scheme 1:**
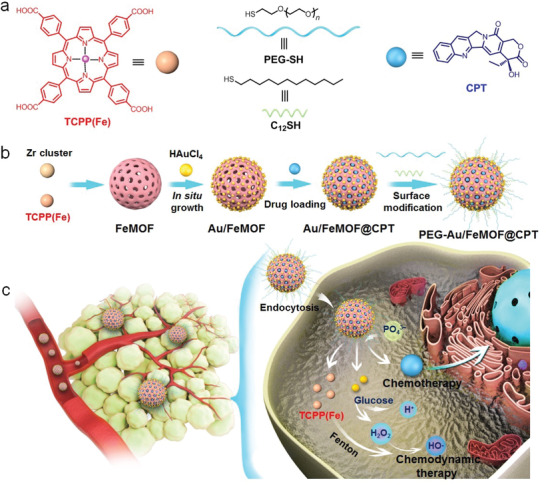
a) The cartoon illustration and chemical structures of the building blocks (**TCPP(Fe)**, **PEG‐SH**, **C_12_SH**, and **CPT**). b) Preparation of the hybrid nanomedicine **PEG‐Au/FeMOF@CPT**. c) High tumor accumulation of **PEG‐Au/FeMOF@CPT** NPs via passive targeting and subsequently cancer cell uptake. Triggered by the intracellular phosphate, the chemotherapeutic drug CPT is released and the cascade catalytic reactions are activated. H_2_O_2_ generated through the oxidation of glucose by Au NPs acts as chemical fuel for Fenton reaction to produce highly toxic ROS to realize chemo/chemodynamic therapy.

**FeMOF** NPs were first prepared through a solvothermal method in dimethylformamide at 95 °C for 5 h. Monodispersed nanoparticles with the diameter around 50 nm were observed in transmission electron microscopy (TEM) image (**Figure** **1**a and Figure S1, Supporting Information). **Au/FeMOF** NPs were further fabricated using **FeMOF** NPs as platforms by directly reducing HAuCl_4_ in water using sodium borohydride as a reductant. As shown in Figure 1b,d, dark spots corresponding to the Au NPs ≈5 nm in diameter were observed on the exterior surface of **Au/FeMOF** NPs. Zeta potential of **FeMOF** NPs decreased from 33.1 to −46.5 mV upon formation of **Au/FeMOF** NPs (Figure S2, Supporting Information). Compared with **FeMOF** NPs, the absorption of **Au/FeMOF** NPs in the range of 500–700 nm remarkably increased (Figure 1f). The reason was that the distance between the Au NPs was close enough, thus resulting in the plasmonic coupling among the particles. Brunauer–Emmett–Teller surface area characterization demonstrated that the crystallinity of MOF scaffold in the hybrid composite was effectively maintained after the formation of **Au/FeMOF** NPs with a surface area as high as 1451 m^2^ g^−1^ (Figure S3, Supporting Information). Additionally, the pore size distribution was measured to be 1.7 nm, confirming that the MOF NPs were able to load anticancer drugs (Figure S4, Supporting Information). Indeed, CPT, a topoisomerase I inhibitor, could be successfully encapsulated into the porous **Au/FeMOF** nanoparticles with a loading content of 7.7% mainly driven by the *π*–*π* stacking interaction between CPT and **TCPP(Fe)** and the coordination between Zr and the quinine group of CPT. UV‐vis spectrum confirmed the co‐existence of the specific absorption corresponding to **Au/FeMOF** nanoparticles and CPT (Figure 1f). As evidenced by TEM and dynamic light scattering (DLS) measurements (Figures S5 and S6, Supporting Information), the drug encapsulation did not change the size and morphology of the hybrid nanoparticles.

**Figure 1:**
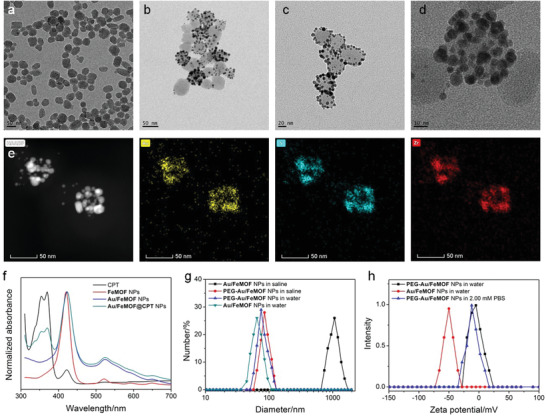
TEM images of a) **FeMOF** NPs, b) **Au/FeMOF** NPs, and c) **PEG‐Au/FeMOF** NPs. d) Enlarged TEM image of **PEG‐Au/FeMOF** NPs. e) EDS elemental mapping images of **PEG‐Au/FeMOF** NPs. f) UV‐vis spectra of CPT, **FeMOF**, **Au/FeMOF**, and **Au/FeMOF@CPT NPs**. g) DLS results of **Au/FeMOF** NPs and **PEG‐Au/FeMOF** NPs in water or saline. h) Zeta potential of **Au/FeMOF** and **Au/FeMOF@CPT NPs** in water or PBS (2.00 × 10^−3^ m).

In order to further improve the colloidal stability of **Au/FeMOF** NPs and block the catalytic capability of the anchored Au NPs in physiological environment, **C_12_SH** and **PEG‐SH** were employed to modify **Au/FeMOF** NPs via Au—S bond. The hydrophobic alkyl chains from **C_12_SH** provided a protective shell to prevent the penetration of phosphate ion and glucose, potentially preserving the nanostructure and silencing the catalytic reaction. On the other hand, PEGylation of **Au/FeMOF** NPs facilitated to enhance their dispersion ability and avoid the absorption by the proteins in the bloodstream. TEM image confirmed that the morphology of the hybrid nanomaterials was maintained after this modification (Figure 1c,d). Energy dispersive spectroscopy (EDS) elemental mapping analyses confirmed the existence of each element (Au, S, Fe, and Zr) in the **PEG‐Au/FeMOF** NPs, providing a direct evidence for the successful fabrication of the hybrid nanomaterial (Figure 1e and Figure S7, Supporting Information). DLS measurements suggested a slight increase in the average diameter after this noncovalent modification (Figure 1g). It should be pointed out that **Au/FeMOF** NPs formed large aggregates in saline. This phenomenon was inhibited by the PEGylation (Figure 1g), indicating that the surface modification was favorable to enhance their dispersion in physiological solution. Zeta potential of the PEGylated **Au/FeMOF** NPs was determined to be −2.4 mV, while the neutral charge was favorable to prolong circulation time and enhance cellular endocytosis of NPs (Figure 1h and Figure S2, Supporting Information).^[^ ^2i^ ^]^ More importantly, this surface modification significantly enhanced the stability of **PEG‐Au/FeMOF** NPs in phosphate buffered saline (PBS). As shown in TEM images, the MOF structures of **FeMOF** and **Au/FeMOF** completely collapsed after culturing in 2.00 × 10^−3^ m of PBS (**Figure** **2**b,e). The fast release of porphyrin ligands shown in Figure 2k also provided convincing evidence that modification was required due to the stability of the **FeMOF** and **Au/FeMOF**. In contrast, the nanostructure of **PEG‐Au/FeMOF** NPs could be maintained for more than 1 day (Figure 2h,k). It should be noted that the drug loading contents for **Au/FeMOF** NPs and **PEG‐Au/FeMOF** NPs (7.1%) were comparable, suggesting that the decoration of Au NPs and PEGylation had negligible impact on drug encapsulation. Moreover, the resultant nanomedicine (**PEG‐Au/FeMOF@CPT** NPs) exhibited excellent dispersion in saline solution, PBS (2 × 10^−3^ m) containing 10% fetal bovine serum and culture medium for 48 h (Figure S8, Supporting Information), making them suitable for potential biomedical applications.

**Figure 2:**
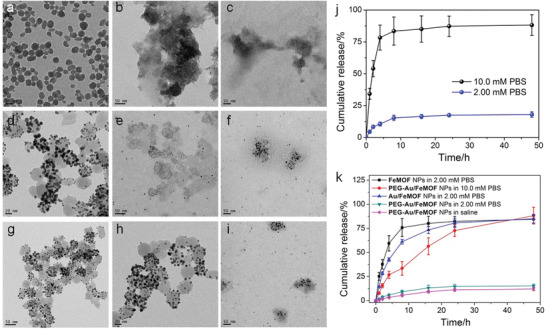
TEM images of a) **FeMOF** NPs, d) **Au/FeMOF** NPs, and g) **PEG‐Au/FeMOF** NPs in water. TEM images of b) **FeMOF** NPs, e) **Au/FeMOF** NPs, and h) **PEG‐Au/FeMOF** NPs in PBS (2.00 × 10^−3^ m). TEM images of c) **FeMOF** NPs, f) **Au/FeMOF** NPs, and i) **PEG‐Au/FeMOF** NPs in PBS (10.0 × 10^−3^ m). j) Release profiles of gold from **PEG‐Au/FeMOF** NPs in PBS containing different phosphate concentrations. k) Release profiles of the porphyrin ligands from **FeMOF** NPs, **Au/FeMOF** NPs, or **PEG‐Au/FeMOF** NPs under different conditions. In (j,k), data represent mean ± s.d. from four independent replicates.

The phosphate‐responsiveness of **PEG‐Au/FeMOF** NPs was further studied in the solution containing much higher phosphate concentration (10.0 × 10^−3^ m). Figure 2k indicated that negligible release of **TCPP(Fe)** was detected in saline, and only little amount (12.2%) of **TCPP(Fe)** was leaked from **PEG‐Au/FeMOF** NPs in the presence of PBS (2.00 × 10^−3^ m) within 48 h. In contrast, 84.5% and 85.3% of **TCPP(Fe)** were released from the naked **FeMOF** NPs and **Au/FeMOF** NPs in PBS (2.00 × 10^−3^ m) after 24 h incubation, respectively. These results indicated that the hybridization of **FeMOF** NPs by Au NPs and subsequent surface modification by **C_12_SH** and **PEG‐SH** significantly improved the stability of **PEG‐Au/FeMOF** NPs. Interestingly, the release speed of **TCPP(Fe)** was significantly accelerated by incubating **PEG‐Au/FeMOF** NPs in PBS containing 10.0 × 10^−3^ m phosphate ions (Figure 2k). TEM image (Figure 2i) revealed that the nanoparticulate structure of **PEG‐Au/FeMOF** NPs was totally collapsed, and the detached Au NPs were well dispersed. The reason was that the coordination interactions between Zr and phosphate ions at high concentration degraded the MOF platform. Notably, phosphate‐sensitive release of CPT was also confirmed. The decreased solution pH protonated the carboxylate groups of **TCPP(Fe)** and quinine group of CPT, accelerating the dissociation of MOF platform and CPT release. The nanomedicine was stable in saline and PBS with low concentration of phosphate (2.00 × 10^−3^ m), while the release rate was dramatically increased in PBS containing 10.0 × 10^−3^ m of phosphate, confirming that the loaded drug could be completely released after cellular internalization by the cancer cells (**Figure** **3**a).

**Figure 3:**
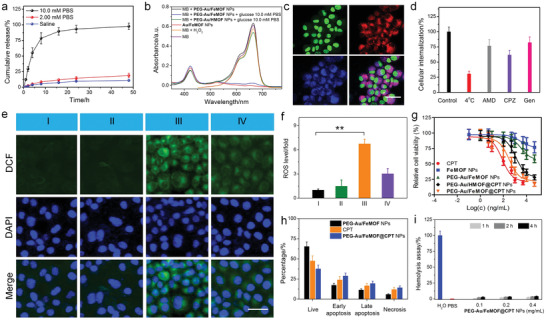
a) Cumulative release curves of CPT from **PEG‐Au/FeMOF@CPT** NPs in solutions containing different concentrations of phosphate ions. b) UV‐vis spectra of MB solution after reaction with different formulations. c) CLSM images of HepG2 cells incubated with **PEG‐Au/FeMOF@CPT** NPs for 8 h. Green fluorescence indicates nucleus from Syto‐9; red fluorescence is attributed to **TCPP(Fe)**; blue fluorescence is attributed to CPT. Scale bar is 50 µm. d) Cellular internalization of **PEG‐Au/FeMOF** NPs under different conditions. e) CLSM images of HepG2 cells stained with DCF and f) quantitative determination of intracellular ROS changes in different groups: I, control; II, **FeMOF** NPs; III, **PEG‐Au/FeMOF** NPs; IV, **PEG‐Au/HMOF** NPs. Green fluorescence reflects the ROS level. Scale bar is 50 µm. g) MTT assay of HepG2 cells after 24 h incubation with CPT, **FeMOF** NPs, **PEG‐Au/FeMOF** NPs, **PEG‐Au/HMOF@CPT** NPs, or **PEG‐Au/FeMOF@CPT** NPs. h) Cell apoptosis determined by Annexin‐V/PI analyses after 48 h incubation. i) Hemolysis assay of **PEG‐Au/FeMOF** NPs at various concentrations. In (a,d,f,h,i), data represent mean ± s.d. from four independent replicates. In (g), data represent mean ± s.d. from six independent replicates. *p* Values were calculated using one‐way analysis of variance (ANOVA) with Tukey's honest significant difference post hoc test (**p < 0.01).

In this hybrid nanomedicine, cascade reactions took place to use glucose as chemical fuel to produce highly toxic radicals. Au NPs can act as GOx‐like enzyme to oxidize glucose to produce H_2_O_2_ and gluconic acid.^[^ ^10^ ^]^ The resultant H_2_O_2_ was further catalyzed by **TCPP(Fe)** to generate OH· through Fenton reaction. The generation of H_2_O_2_ and gluconic acid (reflected by pH) from the first oxidation reaction was dependent on the concentration of glucose. The reaction quickly reached an equilibrium within 6 h (Figures S9 and S10, Supporting Information), which confirmed the high catalytic efficiency of **PEG‐Au/FeMOF** NPs. In the second reaction, methylene blue (MB) was employed as an indicator, which was decomposed by the generated OH· accompanied with the disappearance of blue color. As shown in Figure 3b, no apparent changes in the MB absorbance at 500−700 nm were detected for MB solution incubated with **PEG‐Au/FeMOF** NPs alone. Upon addition of glucose, the degradation of MB by **PEG‐Au/FeMOF** NPs increased along with enhanced phosphate concentration (Figure S11, Supporting Information). The reason was that the oxidation of glucose by **PEG‐Au/FeMOF** NPs under phosphate‐free environment was effectively blocked due to the surface modification, arising from the deactivation of catalytic ability and inhibition of the glucose penetration. Another possible reason was that the integrity of the MOF structure also diminished the Fenton reaction. Interestingly, the decomposition rate of MB was remarkably accelerated using the solution containing high phosphate concentration (10.0 × 10^−3^ m), which was a convincing evidence that the collapse of the hybrid activated the cascade catalytic reactions (Figure 2b and Figure S11, Supporting Information). It should be noted that negligible changes in MB absorption were monitored by replacing **TCPP(Fe)** by *meso*‐tetra(4‐carboxyphenyl)porphine chloride (**HTCPP**) during the preparation of **PEG‐Au/HMOF** NPs, emphasizing that **TCPP(Fe)** acted as the catalyst in the hybrid NPs. Compared with free FeCl_3_ with an extremely high reactivity, a sustained decomposition of MB was observed (Figure S11, Supporting Information). This property was extremely important in biomedical application that could inhibit the catalytic reactions during circulation, thus avoiding side effect toward normal tissues. The phosphate level in extracellular/plasma fluid is about 2.00 × 10^−3^ m, while this number can reach as high as 10.0 × 10^−3^ m inside cells.^[^ ^11^ ^]^ Considering the difference in extracellular and intracellular phosphate concentrations, this nanomedicine guaranteed its safety during delivery process, while efficiently released the loaded drug and activated the cascade catalytic capability after cellular uptake by cancer cells.

With the nanomedicine in hand, the cellular internalization was studied by tracing the intrinsic red fluorescence of **TCPP(Fe)** using HepG2 cells, a human liver cancer cell line. Incubating the cells with **PEG‐Au/FeMOF@CPT** NPs for 8 h resulted in the appearance of strong red fluorescence in cytoplasm (Figure 3c). Confocal laser scanning microscopy (CLSM) image also showed that the blue fluorescence derived from CPT homogenously diffused in entire cells. Quite a large portion of CPT had penetrated into the nucleus as evidenced by the overlap of the blue fluorescence and green fluorescence from the nucleus stained by Syto‐9 dye. Additionally, the time‐dependent endocytosis of **PEG‐Au/FeMOF@CPT** NPs by HepG2 cells was further tracked by detecting the intracellular Au and Zr amount using inductively coupled plasma mass spectrometry (ICP‐MS). Figure S12 in the Supporting Information confirms the co‐existence of Au and Zr in HepG2 cells and the amount increased progressively by extending the culture time.

The endocytic pathways of **PEG‐Au/FeMOF@CPT** NPs were further investigated using ICP‐MS by pre‐treating HepG2 cells with different pharmaceutical inhibitors for endocytosis (Figure 3d). The cellular uptake of the nanomedicine was significantly inhibited at 4 °C, which meant that the endocytosis was energy‐dependent. A 38.6%, 17.9%, and 23.4% reduction in internalization was observed by pre‐treating the cells with chlorpromazine (CPZ), genistein (Gen), or amiloride‐HCl (AMD), respectively, indicating that the endocytosis was mainly through clathrin‐mediated endocytosis, caveolae‐mediated endocytosis, and macropinocytosis pathway.^[^ ^12^ ^]^

Before evaluating the anticancer efficacy, the feasibility of Fenton reaction in the cells was checked. 2,7‐Dichlorofluorescin diacetate (DCFH‐DA) was chosen as a fluorescent probe to detect the generation of ROS in HepG2 cells, whose green fluorescence was waked up after oxidization by OH·. CLSM images in Figure 3e indicated that minor changes in green fluorescence were found for the cells incubated with **FeMOF** NPs. The reason was that the endogenous H_2_O_2_ was insufficient for Fenton reaction to generate a highly reactive OH·. About 6.74‐fold enhancement of ROS was achieved by treating the cells with **PEG‐Au/FeMOF** NPs (Figure 3f), validating the ROS level was significantly elevated through the oxidation of glucose followed by a Fenton reaction. The intracellular production of ROS was greatly inhibited by removing the iron catalyst using **PEG‐Au/HMOF** NPs as indicated by the low green fluorescence in CLSM images, further verifying the cascade catalysis involving the generation of OH·.

3‐(4,5‐Dimethylthiazol‐2‐yl)‐2,5‐diphenyltetrazolium bromide (MTT) assay was adopted to evaluate the anticancer efficacy of the combination chemo/chemodynamic therapy against HepG2 cells (Figure 3g). In the case of **FeMOF** NPs, negligible cytotoxicity was recorded even when the concentration was higher than 50 µg mL^−1^. For chemotherapy alone, the half‐maximal inhibitory concentration (IC_50_) of CPT was calculated to be 206 ± 22 × 10^−9^ m. The cascade starvation and chemodynamic therapy (**PEG‐Au/FeMOF** NPs) effectively inhibited the cell proliferation with an IC_50_ value of 3.51 ± 0.26 µg mL^−1^. Excitingly, the cells treated with chemotherapy and chemodynamic therapy (**PEG‐Au/FeMOF@CPT** NPs) resulted in the lowest IC_50_ value (0.31 ± 0.04 µg mL^−1^). Notably, the IC_50_ value of **PEG‐Au/HMOF@CPT** NPs was much higher than that of **PEG‐Au/FeMOF@CPT** NPs, amplifying that the chemodynamic effect of this hybrid nanomedicine played a pivotal role in its anticancer behavior. According to the MTT data, the combination index was calculated to be 0.41, which confirmed that the combination therapy had an excellent synergy. Moreover, the synergetic anticancer efficacy of **PEG‐Au/FeMOF@CPT** NPs was studied using Annexin‐V/PI dual‐staining. By quantitatively distinguishing the cells in different apoptotic/necrotic status, the chemo/chemodynamic therapy resulted in a large portion of apoptosis (48.7%) and necrosis (14.6%), further confirming the excellent anticancer efficacy of **PEG‐Au/FeMOF@CPT** NPs.

Prior to the antitumor studies, the in vivo safety and pharmacokinetic behaviors of the nanomedicine should be carefully evaluated. As shown in Figure 3i, treatment of **PEG‐Au/FeMOF@CPT** NPs for different time did not cause apparent hemolysis in the test concentration range. The great blood compatibility shall be attributed to the PEGylation of the nanomedicine and subsequently avoid the damage of the membrane of the red blood cells.^[^ ^13^ ^]^

The circulation half‐life of **PEG‐Au/FeMOF@CPT** NPs was determined using ICP‐MS by measuring the Zr amount in blood different time post intravenously (i.v.) injection (Figure S13, Supporting Information). Different from free CPT that was rapidly cleared from the blood with a circulation half‐life of 0.35 ± 0.05 h, the circulation half‐life of **PEG‐Au/FeMOF@CPT** NPs was calculated to be 4.27 ± 0.36 h, which was about 12.2‐fold of free CPT. Additionally, **PEG‐Au/FeMOF@CPT** NPs had much larger total area under the curve than that of CPT, further confirming that the circulation time of **PEG‐Au/FeMOF@CPT** NPs was significantly improved by taking advantage of nanotechnology. More importantly, the Au/Zr ratio in blood at different time post injection almost kept constant (Figure S14, Supporting Information), this meant that the nanomedicine was stable during circulation mainly attributing to the hybridization and surface modification. This property was extremely important to specifically activate the anticancer efficacy in cancer cells while reduce the systemic toxicity of this nanomedicine.

The PEG chains on the surface of **PEG‐Au/FeMOF@CPT** NPs can form a “brush‐like” shell that greatly prevents proteins adsorption, which facilitated to prolong the circulation time and thus promote the accumulation of **PEG‐Au/FeMOF@CPT** NPs in tumor tissue.^[^ ^2i^ ^]^ Using ICP‐MS, the time‐dependent biodistribution of **PEG‐Au/FeMOF@CPT** NPs in the main organs and tumors was quantitatively measured. As shown in **Figure** **4**a, **PEG‐Au/FeMOF@CPT** NPs were highly accumulated in the tumors attributing to the extended circulation time and EPR effect. The intratumoral amount gradually increased from 5.87 ± 0.62 to 7.87 ± 0.68%ID g^−1^ at 12 and 24 h post i.v. injection, and the tumor accumulation of **PEG‐Au/FeMOF@CPT** NPs maintained as high as 6.31 ± 0.57%ID g^−1^ even at 48 h post injection. In sharp comparison, the tumor accumulation of free CPT was quite low and most of the toxic drug stuck in lung (Figure 4b), because the hydrophobic CPT was formed into large aggregations in blood after injection. These differences demonstrated the advantages of nanoformulations in enhancing anticancer efficacy while reducing the side effects of free drug. In vivo fluorescent imaging was also employed to directly visualize the tumor accumulation of **PEG‐Au/FeMOF@CPT** NPs by using the intrinsic fluorescence of the porphyrin ligands (Figure 4c). 24 h post injection, strong signal was observed in tumor tissue, in good agreement with the result obtained from ICP‐MS investigations. CLSM image of the tumor tissue from the mice formulated with **PEG‐Au/FeMOF@CPT** NPs at 24 h post injection further demonstrated that the nanomedicine could deeply penetrate into the tumor, which was important to effectively kill the cancer cells (Figure S15, Supporting Information).

**Figure 4:**
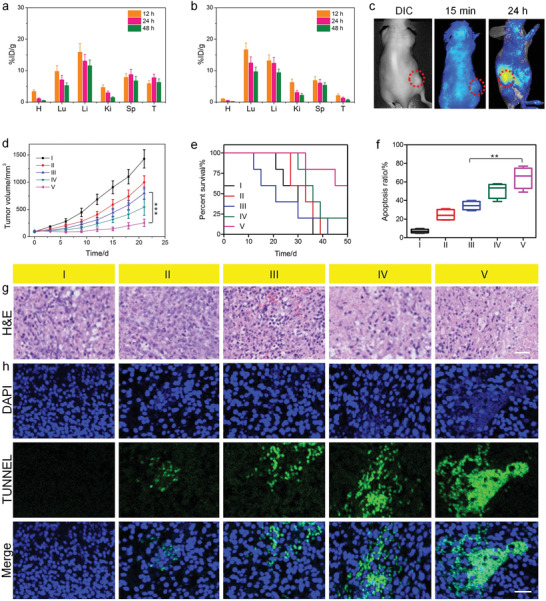
Time‐dependent tissue distributions of CPT for the mice injected with a) **PEG‐Au/FeMOF@CPT** NPs and b) CPT. H, heart; Lu, lung; Li, liver; K, kidney; Sp, spleen; T, tumor. c) Fluorescence imaging of the mice post i.v. injection of **PEG‐Au/FeMOF@CPT** NPs at 15 min and 24 h. d) Tumor growth curves and e) survival ratio of the mice bearing HepG2 tumors received different treatments. f) Apoptosis ratio of the tumor cells in different groups. g) H&E and h) TUNEL staining of the tumor tissues in different groups. I, PBS; II, **PEG‐Au/FeMOF** NPs; III, CPT; IV, **PEG‐Au/HMOF@CPT** NPs; V, **PEG‐Au/FeMOF@CPT** NPs. Scale bars in (g) and (h) are 100 and 50 µm, respectively. Data are expressed as mean ± s.d. In (a,b), data represent mean ± s.d. from four independent replicates. In (d–f), data represent mean ± s.d. from five independent replicates. *p* Values were calculated using one‐way ANOVA with Tukey's honest significant difference post hoc test (**p < 0.01, ***p < 0.001).

With the satisfactory synergistic anticancer performance and high tumor accumulation of **PEG‐Au/FeMOF@CPT** NPs in hand, in vivo antitumor studies were performed using HepG2 tumor‐bearing nude mice. When the tumor volume reached around 100 mm^3^, the mice were divided randomly into five groups and injected with PBS, CPT, **PEG‐Au/FeMOF** NPs, **PEG‐Au/HMOF@CPT** NPs, and **PEG‐Au/FeMOF@CPT** NPs, respectively. Compared with the mice treated with PBS, only moderate therapeutic result was observed for the ones treated with CPT and **PEG‐Au/FeMOF** NPs, mainly attributing to the unsatisfactory anticancer effect of single chemotherapy and CDT (Figure 4d). Administration with **PEG‐Au/HMOF@CPT** NPs resulted in relatively higher antitumor efficacy, because the EPR effect facilitated the high tumor accumulation of CPT. The consumption of glucose due to the oxidation mediated by Au NPs also played a role in starvation therapy. By taking advantages of the EPR effect, tumor‐specific drug release, and synergistic anticancer efficacy, the tumor growth was significantly suppressed for the mice treated with **PEG‐Au/FeMOF@CPT** NPs. According to the tumor volume, the inhibition ratio was determined to be 16.7%, 30.1%, 44.3%, 62.7%, and 85.6% for the mice treated with **PEG‐Au/FeMOF** NPs, CPT, **PEG‐Au/HMOF@CPT** NPs, and **PEG‐Au/FeMOF@CPT** NPs, respectively (Figure S16, Supporting Information). Benefiting from the excellent therapeutic performance, the median survival ratio of mice treated with **PEG‐Au/FeMOF@CPT** NPs was significantly extended (Figure 4e), which was much longer than the other formulations. Hematoxylin and eosin (H&E) staining indicated that the formulation of MOF‐Au‐PEG@CPT resulted in the highest level of apoptotic and necrotic cells in tumor tissues (Figure 4g). Transferase‐mediated dUTP nick end‐labeling (TUNEL) staining indicated a 67.1% cell apoptosis in tumor sites from the mice administrated with MOF‐Au‐PEG@CPT, which was the highest among all the groups (Figure 4f,h), demonstrating that the antitumor performance was greatly enhanced when combining chemotherapy and CDT together.

The systemic toxicity was assessed by detecting the changes in body weight of mice treated with these formulations. Figure S17 in the Supporting Information suggested that the administration of free CPT led to an obvious weight loss due to the nonspecific distribution of the toxic drug. The toxicity of CPT was greatly reduced by encapsulating the drug into the sophisticated nanomedicine. During the therapeutic period, no apparent body weight reduction was noticed from the mice received **PEG‐Au/FeMOF@CPT** NPs at the same dosage. Moreover, no evident symptoms of toxic effects were observed for the mice, such as drinking, eating, urination, grooming, activity, and neurological status. H&E staining of the liver and kidney tissues from the mice administrated with **PEG‐Au/FeMOF@CPT** NPs showed that no damage or inflammatory lesion was observed in these organs (Figures S18 and S19, Supporting Information). Blood biochemistry tests further provided evidences for the negligible long‐term potential toxicity of this nanomedicine. Compared with the healthy mice, no obvious changes in the levels of alkaline phosphatase (ALP), alanine aminotransferase (ALT), aspartate aminotransferase (AST), creatinine (CREA), and blood urea nitrogen (BUN) for the mice injected with **PEG‐Au/FeMOF@CPT** NPs, while these parameters were greatly increased for the mice receiving free CPT treatment (Figures S20–S24, Supporting Information). These studies confirmed the low systemic toxicity of this smart nanomedicine benefiting from the nanotechnology.

In conclusion, a smart nanomedicine was prepared for cancer chemo/chemodynamic therapy based on a hybrid nanomaterial, in which Au NPs were grown in situ on the MOF NPs. The highly porous MOF NPs were employed to load CPT and the hybridization strategy effectively increased the stability of the resultant nanocomposites. Interestingly, the Au NPs were able to oxidize the intracellular glucose to produce H_2_O_2_, which was further used to generate highly toxic •OH for cancer chemodynamic therapy. Surface modification guaranteed the stability of this nanomedicine, and the cascade catalytic reactions were inhibited during circulation. Triggered by the high concentration of phosphate ions, the hybrid nanomaterials collapsed after cellular internalization, resulting in the burst release of the loaded drug and activation of the cascade reactions. Attributing to the EPR effect and sophisticated designs of this nanomedicine, the circulation time was greatly prolonged and the tumor accumulation was significantly improved, which were favorable to enhance its antitumor performance and reduce side effects. In vivo studies demonstrated the excellent synergy combining chemotherapy and chemodynamic therapy, effectively suppressing the tumor growth. This interesting work provided new strategies for the fabrication of theranostic hybrid nanomedicines to overcome limitations faced by the existed modalities, which hold promising potential in cancer treatment.

## Conflict of Interest

The authors declare no conflict of interest.

## Supporting information

Supporting Information
