# Supplementary material for: A Nanomedicine Fabricated from Gold Nanoparticles‐Decorated Metal–Organic Framework for Cascade Chemo/Chemodynamic Cancer Therapy
Source: Adv Sci (Weinh). 2020 Jun 14;7(17):2001060. doi: 10.1002/advs.202001060 (PMC7507500; doi:10.1002/advs.202001060)
Supplement: Supplementary file 1 — Supporting Information [file ADVS-7-2001060-s001.pdf]

Supporting information

A Nanomedicine Fabricated from Gold Nanoparticles Decorated Metal Organic Framework for Cascade Chemo/Chemodynamic Cancer Therapy

Yuan Ding, Hao Xu, Chang Xu, Zongrui Tong, Sitong Zhang, Yang Bai, Yining Chen, Qianhui Xu, Liuzhi Zhou, Hao Ding, Zhongquan Sun, Sheng Yan, Zhengwei Mao*, Weilin Wang**

Dr. Y. Ding, H. Xu, C. Xu, S. Zhang, Y. Bai, Y. Chen, Q. Xu, L. Zhou, H. Ding, Z. Sun, Prof. S. Yan, Prof. W. Wang
Department of Hepatobiliary and Pancreatic Surgery, the Second Affiliated Hospital, School of Medicine, Zhejiang University, Hangzhou, Zhejiang 310009, China
Key Laboratory of Precision Diagnosis and Treatment for Hepatobiliary and Pancreatic Tumor of Zhejiang Province, Hangzhou, Zhejiang 310009, China
Research Center of Diagnosis and Treatment Technology for Hepatocellular Carcinoma of Zhejiang Province, Hangzhou, Zhejiang 310009, China
Clinical Medicine Innovation Center of Precision Diagnosis and Treatment for Hepatobiliary and Pancreatic Disease of Zhejiang University, Hangzhou, Zhejiang 310009, China
Clinical Research Center of Hepatobiliary and Pancreatic Diseases of Zhejiang Province, Hangzhou, Zhejiang 310009, China
Emails: wam@zju.edu.cn (W. Wang); shengyan@zju.edu.cn (S. Yan)

Z. Tong, Prof. Z. Mao
MOE Key Laboratory of Macromolecular Synthesis and Functionalization, Department of Polymer Science and Engineering, Zhejiang University, Hangzhou, Zhejiang 310027, China
Key Laboratory of Precision Diagnosis and Treatment for Hepatobiliary and Pancreatic Tumor of Zhejiang Province, Hangzhou, Zhejiang 310009, China
Email: zwmao@zju.edu.cn (Z. Mao)

1. Materials and methods

Zirconyl chloride octahydrate ($ZrOCl_2·8H_2O$), benzoic acid, gold(III) chloride hydrate ($HAuCl_4$, 99.995% trace metals basis), sodium borohydride ($NaBH_4$), 1-dodecanethiol (**$C_{12}SH$**), *N*,*N*-dimethylformamide (DMF) and camptothecin were purchased from Sigma-Aldrich. Iron(III) meso-tetra(4-carboxyphenyl)porphine chloride **TCPP(Fe)** and tetrakis(4-carboxyphenyl)porphyrin (HTCPP) were gained from Fisher Scientific. Thiolated polyethylene glycol (**PEG-SH**, M_w: ~5000 g mol^{-1}) was bought from Biochempeg Scientific Inc. MTT cytotoxicity assay kits for cell viability detection were bought from Thermo Fisher Scientific. Solvents were either employed as purchased or dried according to procedures described in the literature. Millipore ultrapure water was obtained on a Milli-Q purification system. Transmission electron microscopy (TEM) investigations were carried out on a HT-7700 instrument. UV-vis spectra were obtained by a Genesys 10s UV-vis spectrophotometer (Thermo Scientific, Waltham, MA). Fluorescence spectra were collected via a Hitachi F-7000 fluorescence spectrophotometer. Nitrogen adsorption-desorption isotherm measurement was couducted on a Micromeritics ASAP 2020M automated sorption analyzer (Micromeritics Co., USA). The specific surface areas and the corresponding pore size distribution of **Au/FeMOF NPs** were acquired based on the N_2 adsorption isotherm at 77 K by Brunauer−Emmett−Teller (BET) method and the density functional theory (DFT) model, respectively. The high-performance liquid chromatography (HPLC) analysis for determining the release efficiency of CPT from MOF nanostructures was conducted on a Agilent1200 machine (Agilent, USA). The zirconium and Au contents for evaluating the uptake efficiency of tumor cells were monitored using inductively coupled plasma optical emission spectroscopy (ICP-MS). Confocal laser scanning microscopy (CLSM) images was recorded on a Zeiss LSM 780 microscope. H&E tissue and cell staining was performed by BBC Biochemical (Mount Vernon, WA) and the images were collected using a BX41 bright field microscopy (Olympus).

2. Experimental Section

Prepartion of FeMOF NPs. The FeMOF NPs used in this work were synthesized according to a previous report with slight modification.[1] First, $ZrOCl_2·8H_2O$ (300 mg), **TCPP(Fe)** (100 mg) and

benzoic acid (2.2 g) were ultrasonically dissolved in 100 mL of DMF solution. Then, the mixture was heated to 95 ºC for 5 h. With the reaction solution cooled down to room temperature, the formed amaranth **FeMOF** NPs were washed twice with DMF by centrifugation at 15,000 rpm for 15 min to remove excess chemicals. The products were re-dispersed in DMF for storage. To quantify the mass concentration of **FeMOF** NPs, 1 mL of MOF NPs were centrifuged, immersed in acetone and washed with acetone to remove the residual DMF solvent. Finally, the precipitates were dried at 60 ºC and weighed by an analytical balance. Similar method was utilized to prepare **HMOF** NPs by using HTCPP as the ligand.

Synthesis of Au/FeMOF NPs. The **Au/FeMOF** NPs were prepared via in situ growth of Au NPs on the **FeMOF** NPs surface. Briefly, 600 μL of **FeMOF** NPs (35 mg mL^{-1}) in DMF and 1 mL of $HAuCl_4$ (8 mg mL^{-1}) was added into 250 mL of ultrapure water in turn. The resultant mixture was reacted for 10 s under magnetic stirring, followed by rapid addition of 600 μL of freshly prepared $NaBH_4$ solution (3.5 mg mL^{-1}). The formed **Au/FeMOF** NPs were then washed twice with acetone and once with ultrapure water to remove the unreacted chemicals and residual DMF by centrifugation at 8,000 rpm for 15 min. The final particulates were re-dispersed in Millipore ultrapure water.

Synthesis of PEG-Au/FeMOF@CPT NPs. CPT (0.3 mg) was solublized in DMF and the obtained solution was added into **Au/FeMOF** NPs solution (1 mL, 1 mg mL^{-1}). The resultant mixture was stirred for 24 h. After centrifugation and washing, the synthetic **Au/FeMOF@CPT** NPs were isolated and dispersed into DMF for further use. The surface modification was conducted using **C_{12}SH** and **PEG-SH** based on the strong Au-S bond for improved biocompatibility. Briefly, **C_{12}SH** (0.3 mg) and **PEG-SH** (2 mg) was added into **Au/FeMOF@CPT** NPs solution (1 mL, 1.5 mg mL^{-1}), and then the resultant mixture was shakily reacted overnight at room temperature. After centrifugation and washing, the obtained **PEG-Au/FeMOF@CPT** NPs were isolated and dispersed into water for further use. Similar method was used to modify **Au/FeMOF** NPs and **Au/HMOF@CPT** NPs to afford **PEG-Au/FeMOF** NPs and **PEG-Au/HMOF@CPT** NPs. The loading content was calculated by the following equations:

Loading content (%) = $(m_{loaded}/m_{loaded} + m)*100$

m_{loaded} is the mass of CPT encapsulated in the nanohybrids, and m is the mass of the hybrid nanomaterials used for the preparation of nanomedicines.

Phosphate-sensitivity of the hybrid nanomaterials. The stability of the nanomaterials towards phosphate was studied by recording the release profiles of porphyrin ligands and Au NPs as well as monitoring the morphological changes in different solutions. Samples with a normalized MOF weight of 0.15 mg were dispersed into 1.5 mL of PBS (2 mM or 10 mM) or saline, respectively. At predetermined time interval, 100 μL of the solutions were withdrawn and centrifuged at a speed of 15,000 rpm for 15 min. Then the liberated porphyrin ligands in the supernatant were gathered, and recorded the absorbance at 410 nm by a microplate reader. The time-dependent release behaviors were then determined using a standard curve. For the determination of Au release, 100 μL of the solution were taken out and subjected to centrifugation (15,000 rpm, 15 min) at certain time points. After that, 60 μL of Au NPs-containing supernatant was collected and decomposed by aqua regia. Finally, the samples were diluted and filtered for ICP-MS measurements. TEM was also employed to reveal the morphology changes of the nanomaterials before and after PBS treatments. The corresponding nanomaterials were dissolved in PBS buffer (2 mM or 10 mM), respectively. Partial solutions were withdrawn for ultrafiltration at different time points. After washing several times, the residue was resuspended with water and analyzed by TEM.

Drug release measurements. The phosphate-sensitive drug release behaviors of the CPT-loaded nanomaterials were investigated by monitoring the CPT release in various solutions. Typically, the nanomaterials (2 mg) were dispersed into 1.5 mL of saline or PBS (2 mM or 10 mM), respectively. At predetermined time intervals, 100 μL of the solutions were taken out for centrifugation. The supernatants were collected for further HPLC measurements. The drug release rate was then calculated with the formula of standard curve.

Cell Culture. HepG2 cells were cultured in Dulbecco's modified Eagle's medium (DMEM) containing 10% fetal bovine serum (FBS) and 1% penicillin/streptomycin. Cells were detached using trypsin (0.5% *w/v* in PBS) upon confluence. The cells were harvested from the cell culture medium by incubating in a

trypsin solution for 3 min. The cells were centrifuged, and the supernatant was discarded. A 3.0 mL portion of serum-supplemented DMEM was added to neutralize any residual trypsin. The cells were resuspended in serum-supplemented DMEM at a concentration of 1.0 × 10^4 cells/mL. Cells were cultured at 37 °C and 5% CO_2.

Quantification of cellular uptake. For cellular uptake study, HepG2 cells were seeded to 6-well plates at a density of 2 × 10^5 per well and placed in a 37 ºC humidified incubator with 5% CO_2/95% air supply. 24 h later, the cells were treated with the nanohybrids (100 μg mL^{-1} at normalized MOF concentration) for various times. After rinsing several times with PBS, the cells were collected, counted and decomposed by aqua regia. Finally, the solutions were analyzed by ICP-MS following further dilution and filtration.

Confocal fluorescence imaging. HepG2 cells were treated with **PEG-Au/FeMOF@CPT** NPs (the concentration of **TCPP(Fe)** was 5.0 μM) in the culture medium at 37 °C for 8 h. After being washed three times with PBS, the cells were stained with Syto-9 (400 nM) for 45 min. After being washed three times with PBS, the cells were further culture for 1 h. Then, the cells were washed three times with PBS, fixed with fresh 4.0% formaldehyde at room temperature for 15 min, and washed with PBS for two times. The images were taken using a Zeiss LSM 780 confocal laser scanning microscope.

Intracellular ROS Detection. DCFH-DA was employed as a fluorescent ROS probe to indicate CDT-induced oxidative stress. Briefly, HepG2 cells were treated with the PBS, **FeMOF** NPs, **PEG-Au/FeMOF** NPs, or **PEG-Au/HMOF** NPs for 8 h. DCFH-DA (25 µM) was added and the cells were incubated for another 4 h. Then, the medium was removed carefully and the cells washed with PBS three times. The cells were fix by *Z*-fix solution for 15 mins and washed with PBS three times. The nuclei were stained with DAPI. The images were taken using a Zeiss LSM 780 confocal laser scanning microscope.

Evaluation of Cytotoxicity. The cytotoxicity of the different formulations against HepG2 cells was determined by 3-(4′,5′-dimethylthiazol-2′-yl)-2,5-diphenyl tetrazolium bromide (MTT) assays in a 96-well cell culture plate. All solutions were sterilized by filtration with a 0.22 μm filter before tests.

HepG2 cells were seeded at a density of 1.0 × 10^4 cells/well in a 96-well plate, and incubated for 24 h for attachment. Cells were then incubated with the different formulations at various concentrations for 48 h. After washing the cells with PBS buffer, 20 μL of a MTT solution (5 mg/mL) were added to each well. After 4 h of incubation at 37 °C, the MTT solution was removed, and the insoluble formazan crystals that formed were dissolved in 100 μL of dimethylsulfoxide (DMSO). The absorbance of the formazan product was measured at 570 nm using a spectrophotometer (Bio-Rad Model 680). Untreated cells in media were used as a control. All experiments were carried out with five replicates.

Analyses of the endocytotic pathways. The evaluation of endocytotic pathways was performed as follows. HepG2 cells were seeded at a density of 5.0 × 10^5 cells/well in 6-well cell culture plates. The cells were left to grow for 24 h in DMEM media containing 10% FBS at 37 °C in 5% CO_2 atmosphere. For the inhibition of energy-dependent endocytosis, HepG2 cells were cultured at 4 ^{o}C in the presence of **PTCG NPs** for 3 h. HepG2 cells were treated with different inhibitors including chlorpromazine (30.0 μM), genistein (0.2 mM), or amiloride (1.0 mM) in serum free DMEM medium for 1 h at 37 °C, respectively. Then, **PEG-Au/FeMOF** NPs were further added to the medium for another 3 h incubation. Subsequently, the medium was removed and the cells were washed 3 times using PBS. The intracellular Au amount was determined using ICP-MS. HepG2 cells treated with **PEG-Au/FeMOF** NPs in the absence of inhibitors at 37 °C for 3 h was used as a control. Percent internalization was normalized to the control group in the absence of inhibitors. All experiments were carried out with four replicates.

Determination of the percentage of apoptotic cells at different stages. HepG2 cells were seeded in 6-well cell culture plates (5.0 × 10^5 cells/well). After 12 h incubation, the medium was replaced by fresh growth media containing CPT (100 nM), **PEG-Au/FeMOF** NPs (0.45 μg/mL) or **PEG-Au/FeMOF** NPs (the amount of CPT was 100 nM). The cells were incubated for 24 h and harvested with EDTA-free trypsin (0.25%). Then the cells were carefully washed by PBS and stained by annexin-V FITC and propidium iodide (PI) according to the manufacturer's protocol. Flow cytometry was performed and data were analyzed. The cells without any treatment were utilized as a control.

Evaluation of Hemolysis. Blood was collected from the nude BALB/c mice. The blood sample was diluted 10 times with PBS and centrifuged at 1500 rpm for 10 min. The centrifuged sample was washed with sterile PBS for five times by centrifugation and suction to isolate erythrocytes. The concentration of the resultant blood cells was adjusted to 2% (*v*/*v*). An 100 μL sample solution was added to the blood cells (1000 μL) and the mixture was incubated for different time at 37 ^{o}C. The corresponding sample was centrifuged for 15 min at 2000 rpm. In order to assess the hemolytic activity, the released haemoglobin in supernatant was determined by measuring the absorbance at 541 nm. The percentage of hemolysis was determined as $(A_{sample} - A_0)/(A_{100} - A_0) * 100\%$, where A_{sample}, A_{100}, and A_0 is the absorbance of the sample, the completely lysed red blood cells in distilled water, and zero hemolysis in PBS. All hemolysis assays were conducted with three replicates.

Animals and Tumor Models. Female nude mice (4 weeks old, ~20 g body weight) were purchased from Zhejiang Academy of Medical Sciences and maintained in a pathogen-free environment under controlled temperature (24 °C). All animal experiments were carried out in accordance with the Institute of Laboratory Animal Resources guidelines. Ethical approval (No. 2018-285) was granted by the Institutional Animal Care and Use Committee of Zhejiang Academy of Medical Sciences, China. The female nude mice were injected subcutaneously in the right flank region with 200 μL of cell suspension containing 5×10^6 HepG2 cells. The tumors were allowed to grow to ~100 mm^3 before experimentation. The tumor volume was calculated as (tumor length) × (tumor width)^2/2.

Pharmacokinetics and Biodistribution. For pharmacokinetic studies, the mice were randomly divided into two groups (n = 3) and *i.v.* injected with CPT (2.0 mg/kg) or **PEG-Au/FeMOF@CPT** NPs (5.0 mg/kg). The blood samples (0.1 mL) were taken from retro-orbital sinus at desired time after injection, and the blood samples were mixed with physiological buffered saline (PBS, 900 μL) containing 10.0 mM EDTA anticoagulant. All blood samples were centrifuged for 10 min at 10000 rpm to isolate the plasma. Proteins were precipitated upon addition of acetonitrile (120 μL) and incubation for 3 h at 4 ^{o}C. The amount of CPT and Au (or Zr) in the resulting supernatant was measured using HPLC and ICP-MS, respectively. For biodistribution analysis, the mice were *i.v.* injected with CPT (2.0 mg/kg) or **PEG-**

Au/FeMOF@CPT NPs (5.0 mg/kg). The main organs (heart, liver, lung, spleen and kidney) and tumor tissues were excised at 12, 24, and 48 h post-injection and kept in dry ice. Organs were digested using concentrated nitric acid for ICP-MS.

For the determination of CPT, the tissues were harvested, weighed, and placed in a bead-containing homogenizer tubes. 100 μL of HCl (0.1 N) and 400 μL of lysis reagent were added to the tubes. Then, the tissues were homogenized by using a homogenizer for 60 s at a speed of 5 m s^{-1}. This process was repeated 15 times at 5-min intervals for each sample. After every other homogenization procedure, the tissue samples were cooled on ice. Following completion of the 15-cycle homogenation process, the homogenate was centrifuged at 14,000 rpm at 10 ^{o}C for 30 min. Cold methanol (800 μL) was added to the supernatant (200 μL) to precipitate the proteins. The mixed solution was centrifuged at 14,000 rpm at 10 ^{o}C for 15 min. To measure the amount of free CPT in each tissue, the supernatant solution (100 μL) from the centrifuged solution was mixed with 100 μL of HCl (0.1 N). The resulting solution was injected into a reverse phase HPLC for measurements.

***In Vivo* Antitumor Efficacy Evaluation.** The nude mice were randomly divided into six groups (n = 5) when the tumor volume reached around 100 mm^3, and *i.v.* injected with PBS, CPT (1.00 mg/kg), **PEG-Au/FeMOF** NPs (13.0 mg/kg), **PEG-Au/HMOF@CPT** NPs (1.00 mg CPT/kg) and **PEG-Au/FeMOF@CPT** NPs (1.00 mg CPT/kg) every 3 days for three times, respectively. Tumor volume and body weight were measured every 3 days. In the histological assay, the liver, kidney and tumor tissues were fixed in 4% paraformaldehyde for 24 h. The specimens were dehydrated in graded ethanol, embedded in paraffin, and cut into 5 mm thick sections. The fixed sections were deparaffinized and hydrated according to a standard protocol and stained with hematoxylin and eosin (H&E) for microscopic observation. Apoptosis of the tumor cells in the mice after treatments was determined by the TUNEL method according to the manufacturer's instructions.

In Vivo Biosafety Analysis. The mice were *i.v.* injected with PBS, CPT (1.00 mg/kg), and **PEG-Au/FeMOF@CPT** NPs (1.00 mg CPT/kg) every 3 days for three times, respectively. The mice were anaesthetized and euthanized at 21 days post injection, and the eyeballs were removed, followed by

collection of blood samples for blood chemistry tests and routine blood analysis. The mice treated with PBS were used as the blank control.

Statistical analysis. Data are presented as the mean ± standard deviation (s.d.) based on at least three independent experiments. Statistical analysis of data was performed with one-way analysis of variance (ANOVA) with Tukey's honest significant difference post-hoc test using GraphPad Prism 6.0 software (GraphPad Software, San Diego, CA). The level of significance was defined at $*p < 0.05$, $**p < 0.01$ and $***p < 0.001$, respectively.

3. Supporting Figures

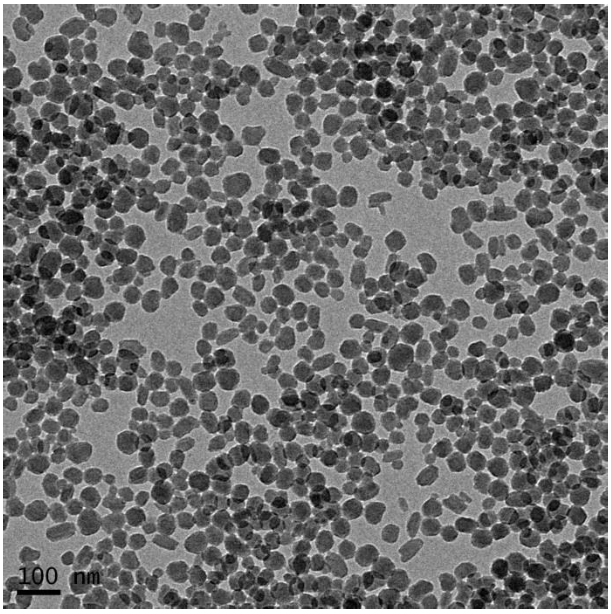

Figure S1. TEM image of **FeMOF** NPs.

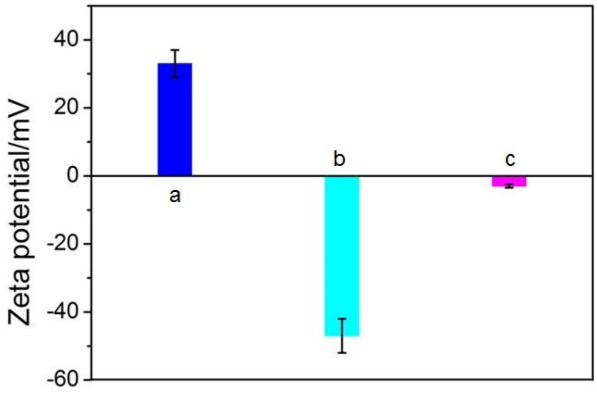

Figure S2. Zeta potential of a) **FeMOF** NPs, b) **Au/FeMOF** NPs and c) **PEG-Au/FeMOF** NPs.

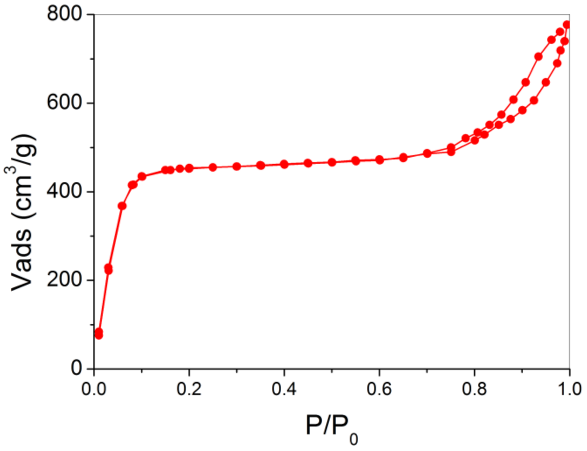

Figure S3. N_2 adsorption-desorption isotherm of **Au/FeMOF** NPs.

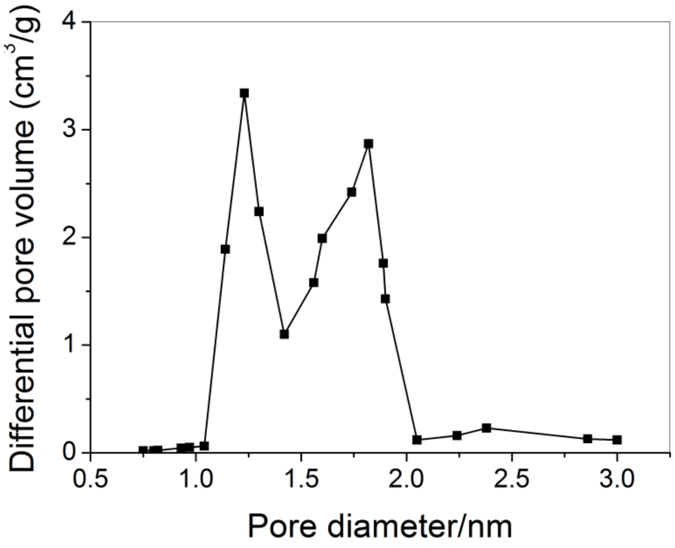

Figure S4. The pore size distribution of **Au/FeMOF** NPs.

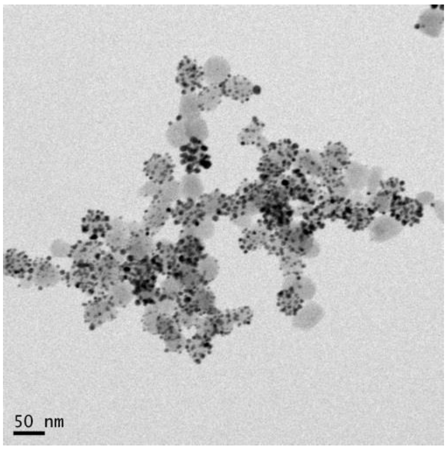

Figure S5. TEM image of **Au/FeMOF@CPT** NPs.

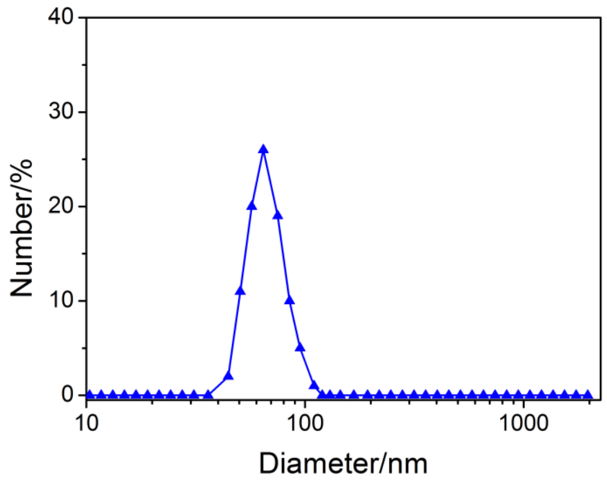

Figure S6. DLS result of **Au/FeMOF@CPT** NPs.

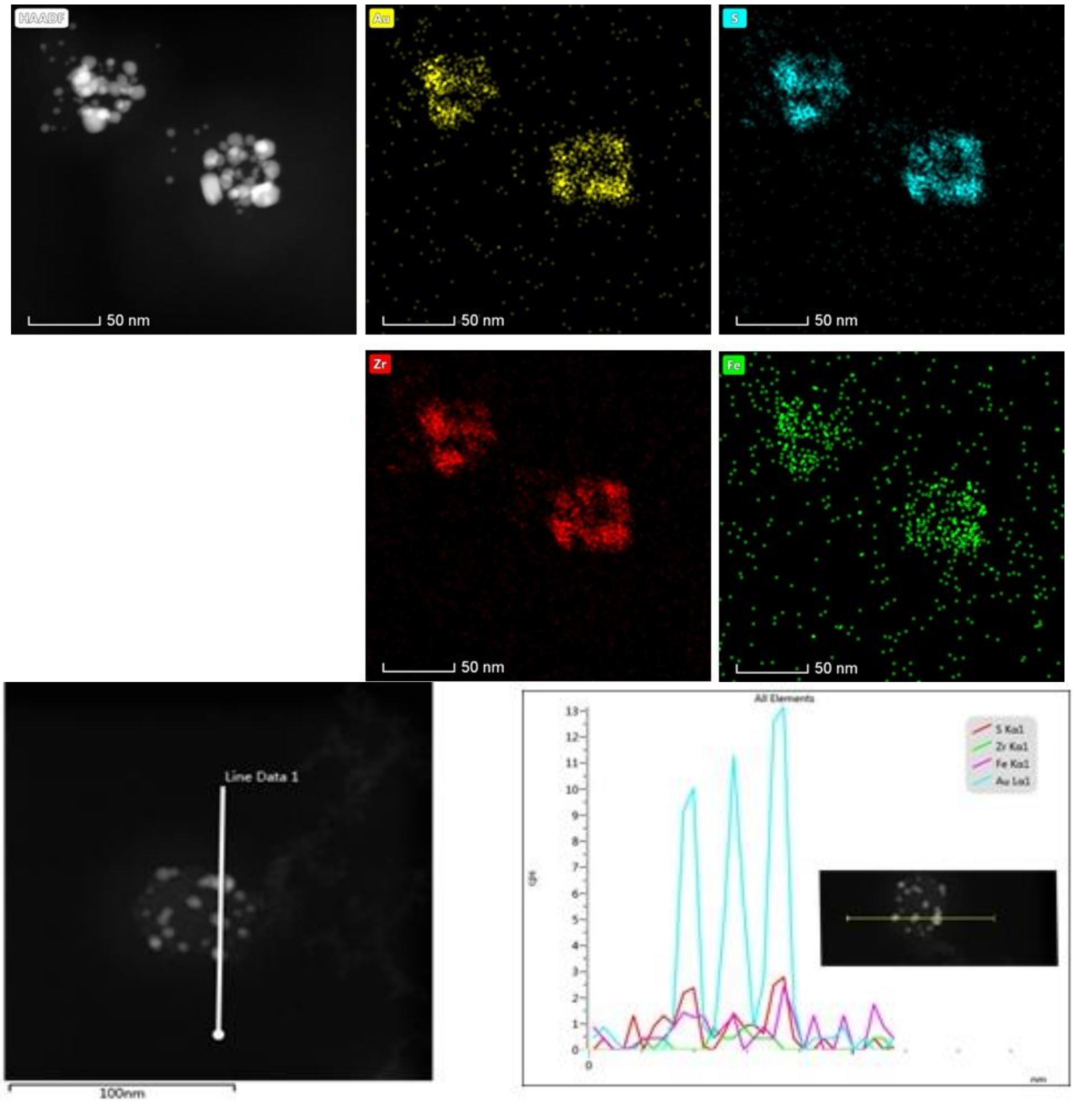

Figure S7. EDS elemental mapping images of **PEG-Au/FeMOF** NPs.

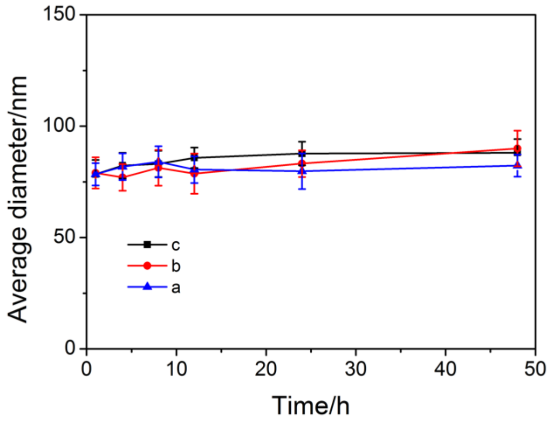

Figure S8. The hydrodynamic diameter variations of **PEG-Au/FeMOF@CPT** NPs during 48 h of dispersion in a) saline, b) PBS (2 mM) containg FBS (10%) and c) cell culture medium, respectively.

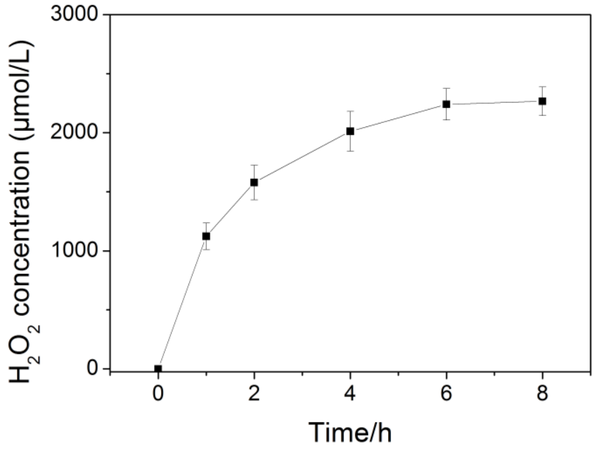

Figure S9. The generated H_2O_2 at different time points arising from the **PEG-Au/FeMOF**-catalyzed decomposition reaction of glucose (2 mg/mL).

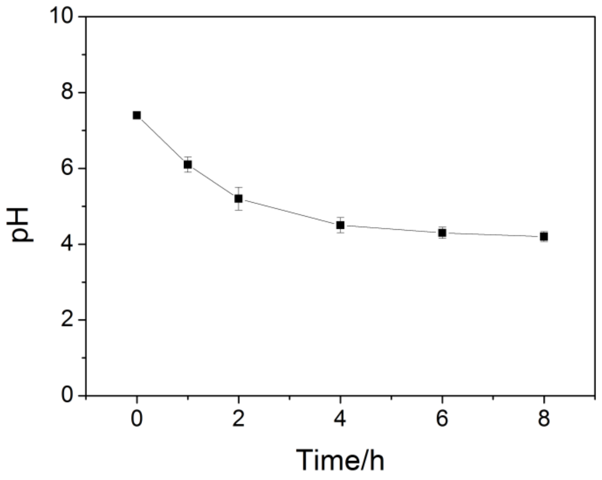

Figure S10. The changes in pH values at different time points arising from the **PEG-Au/FeMOF**-catalyzed decomposition reaction of glucose (2 mg/mL).

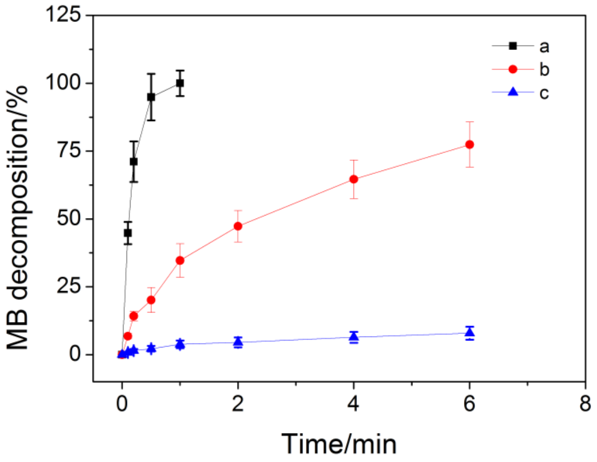

Figure S11. MB decomposition by a) $FeCl_3$ (1.00 μM) + H_2O_2 (200 μM), b) **PEG-Au/FeMOF** NPs in PBS (10.0 mM) containing glucose (5 mg/mL) and c) **PEG-Au/FeMOF** NPs in PBS (2.00 mM) containing glucose (5 mg/mL). The concentration of **TCPP(Fe)** in the **PEG-Au/FeMOF** NPs was 10.0 μM.

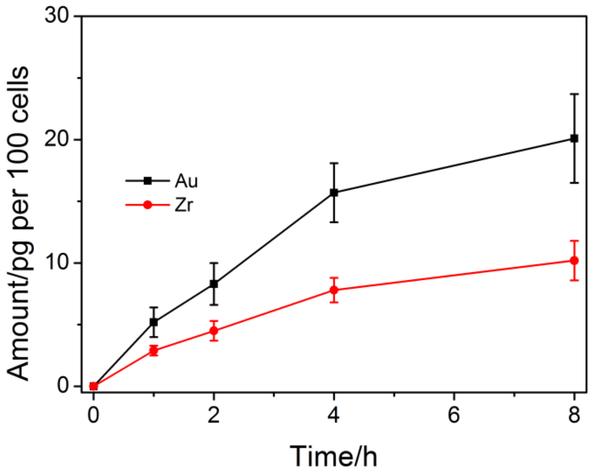

Figure S12. Internalization study of **PEG-Au/FeMOF@CPT** NPs by quantifying intracellular Au and Zr amount.

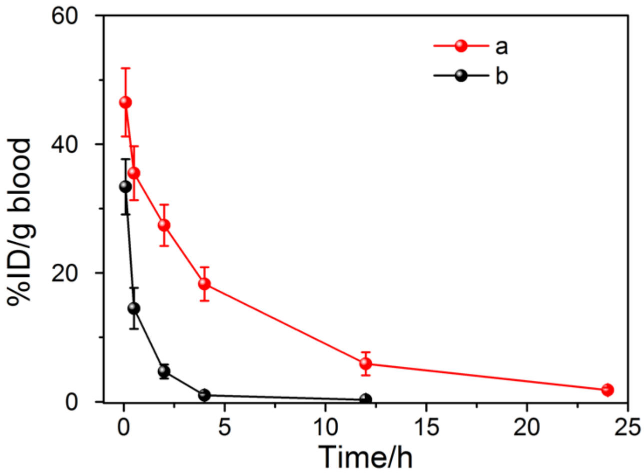

Figure S13. Plasma concentration of a) **PEG-Au/FeMOF@CPT** NPs and b) free CPT versus time after *i.v.* injection.

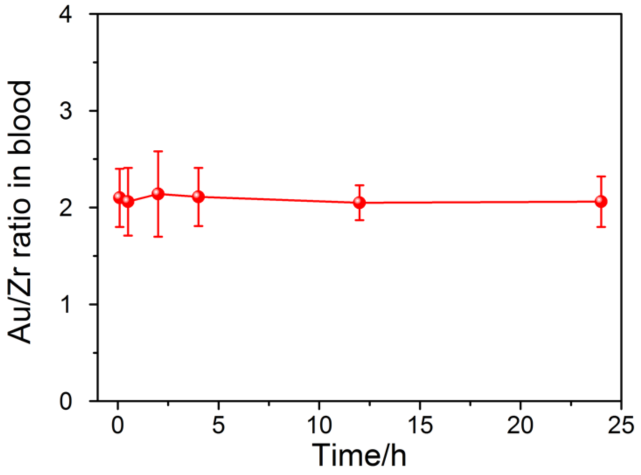

Figure S14. The changes in Au/Zr ratio in plasma versus time after *i.v.* injection of **PEG-Au/FeMOF@CPT** NPs.

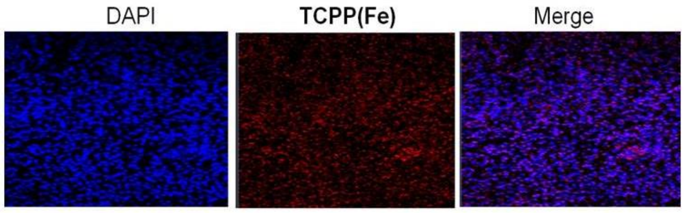

Figure S15. CLSM image of the tumor tissue from the mice treated with **PEG-Au/FeMOF@CPT** NPs.

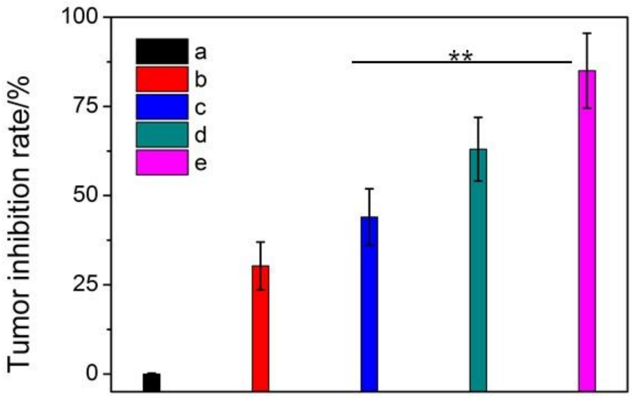

Figure S16. Tumor inhibition rate of the mice treated with a) PBS, b) **PEG-Au/FeMOF** NPs, c) CPT, d) **PEG-Au/HMOF@CPT** NPs and e) **PEG-Au/FeMOF@CPT** NPs, respectively. $**p < 0.01$.

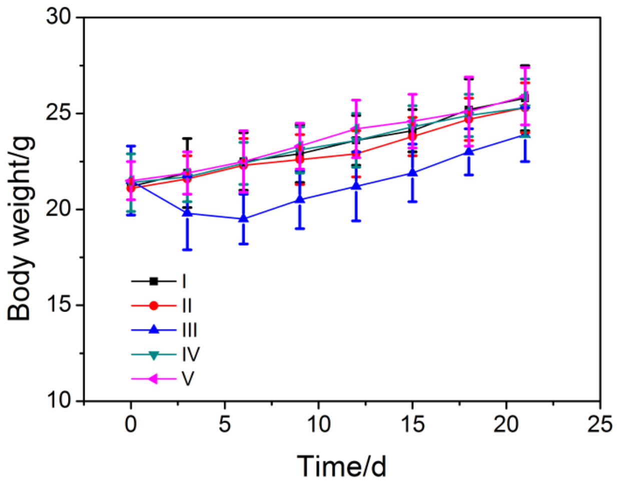

Figure S17. Body weight changes of the mice treated with different formulations: I, PBS; II, **PEG-Au/FeMOF** NPs; III, CPT; IV, **PEG-Au/HMOF@CPT** NPs; V, **PEG-Au/FeMOF@CPT** NPs.

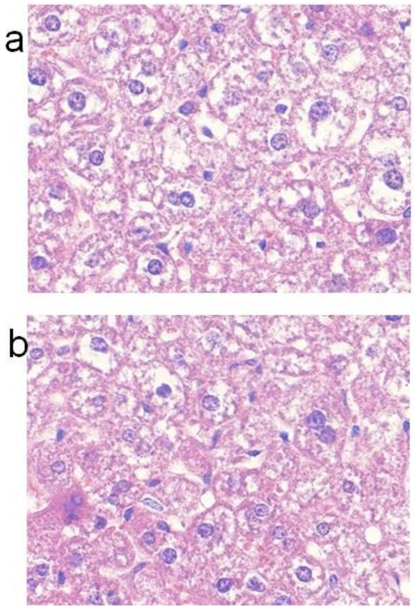

Figure S18. H&E staining of the liver tissues from a) the healthy mouse and b) **PEG-Au/FeMOF@CPT** NPs.

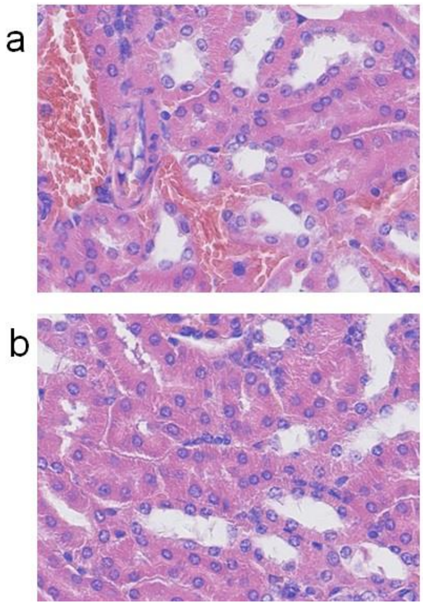

Figure S19. H&E staining of the kidney tissues from a) the healthy mouse and b) **PEG-Au/FeMOF@CPT** NPs.

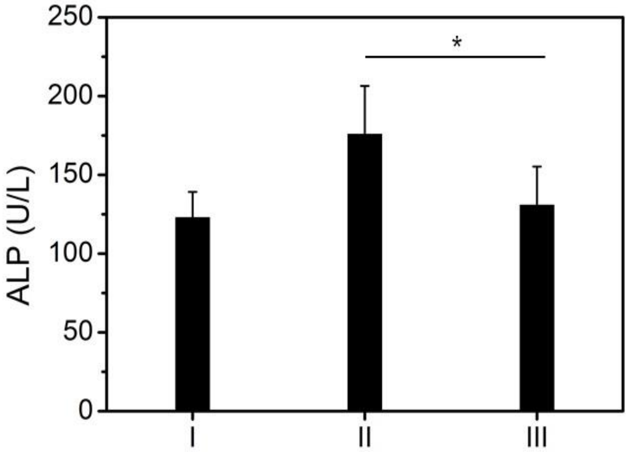

Figure S20. The levels of ALP from the mice treated with I) PBS, II) free CPT and III) **PEG-Au/FeMOF@CPT** NPs. *$p < 0.05$.

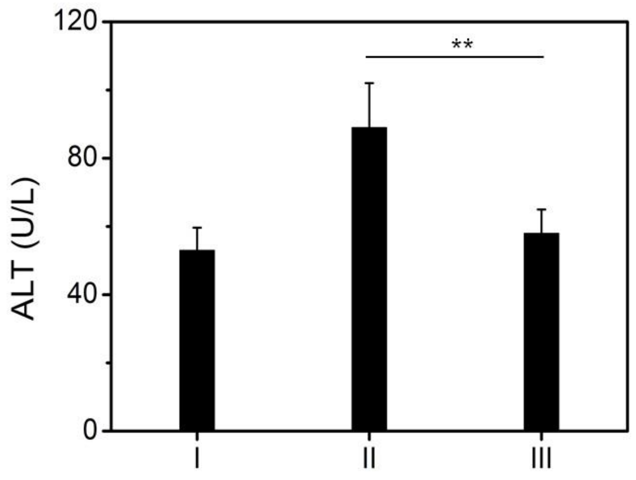

Figure S21. The levels of ALT from the mice treated with I) PBS, II) free CPT and III) **PEG-Au/FeMOF@CPT** NPs. **$p < 0.01$.

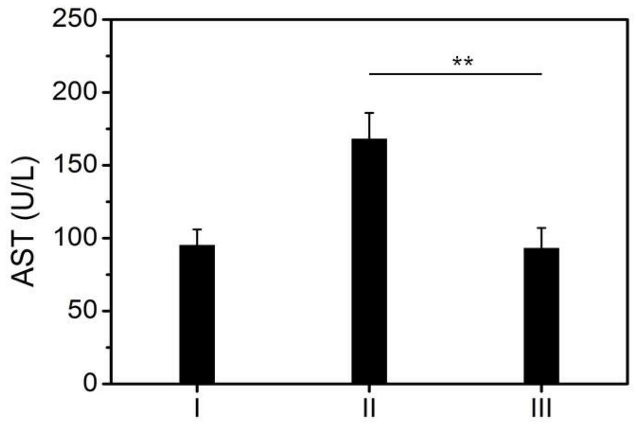

Figure S22. The levels of AST from the mice treated with I) PBS, II) free CPT and III) **PEG-Au/FeMOF@CPT** NPs. **$p < 0.01$.

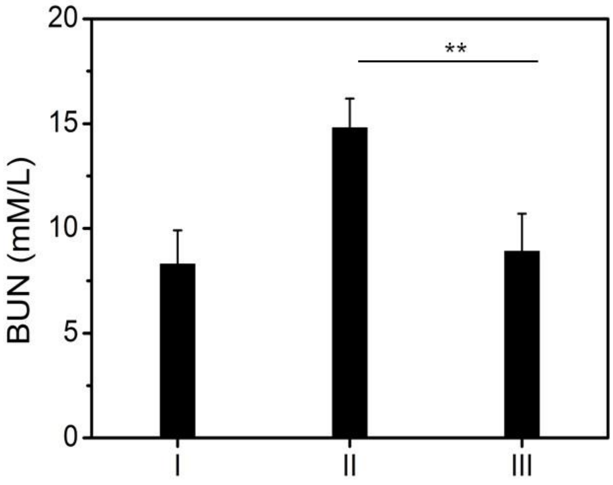

Figure S23. The levels of BUN from the mice treated with I) PBS, II) free CPT and III) **PEG-Au/FeMOF@CPT** NPs. ** indicates $p < 0.01$.

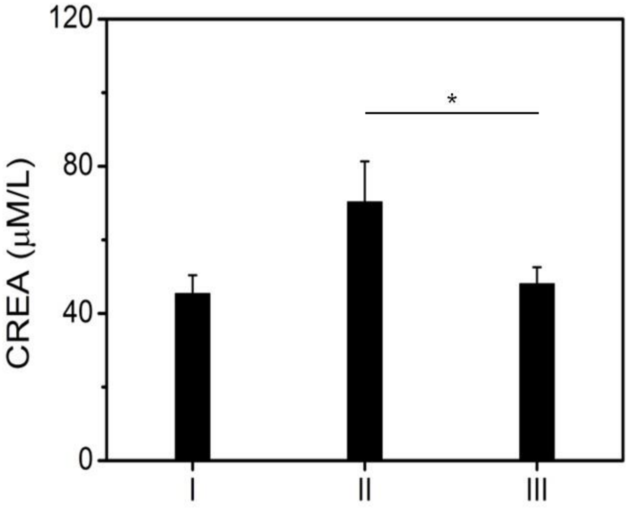

Figure S24. The levels of CREA from the mice treated with I) PBS, II) free CPT and III) **PEG-Au/FeMOF@CPT** NPs. * indicates $p < 0.05$.

4. References

[1] J. Park, Q. Jiang, D. Feng, L. Mao, H.-C. Zhou, *J. Am. Chem. Soc.* **2016**, *138*, 3518.
